# Unexpected genetically determined immune dysregulation with liver involvement: GIMAP5 therapeutic dilemmas between targeted therapy and HSCT

**DOI:** 10.3389/fimmu.2026.1820281

**Published:** 2026-06-10

**Authors:** Mattia Moratti, Michele La Manna, Lucia Colucci, Cristina Cifaldi, Silvia Di Cesare, Gioacchino Andrea Rotulo, Beatrice Rivalta, Donato Amodio, Andrea Pietrobattista, Andrés Caballero-Oteyza, Elisabetta Lembo, Chiara Passarelli, Emma Concetta Manno, Michele Proietti, Gigliola Di Matteo, Giuseppe Palumbo, Caterina Cancrini

**Affiliations:** 1 Pediatric Unit, IRCCS Azienda Ospedaliero-Universitaria di Bologna, University of Bologna, Bologna, Italy; 2 PhD Program in Immunology, Molecular Medicine and Applied Biotechnology, University of Rome Tor Vergata, Rome, Italy; 3 Research Unit of Primary Immunodeficiencies, Bambino Gesu’ Children’s Hospital, IRCCS, Rome, Italy; 4 Department of Pediatric Hemato-Oncology and Cell and Gene Therapy, Bambino Gesù Children’s Hospital, Scientific Institute for Research and Healthcare (IRCCS), Rome, Italy; 5 Residency School of Pediatrics , University of Rome “Tor Vergata”, Rome, Italy; 6 Department of Systems Medicine, University of Rome Tor Vergata, Rome, Italy; 7 Clinical Immunology and Vaccinology Unit, Bambino Gesù Children’s Hospital, IRCCS, Rome, Italy; 8 Metabolic Diseases and Hepatology Unit, Bambino Gesù Children’s Hospital, IRCCS, Rome, Italy; 9 Institute for Immunodeficiency, Center for Chronic Immunodeficiency, University Hospital Freiburg, Freiburg, Germany; 10 RESiST-Cluster of Excellence 2155, Hanover Medical School, Hanover, Germany; 11 Laboratory of Medical Genetics, Translational Cytogenomics Research Unit, Bambino Gesù Children’s Hospital, IRCCS, Rome, Italy; 12 Clinic for Immunology and Rheumatology, Hanover Medical School, Hanover, Germany

**Keywords:** cytopenia, GIMAP5 deficiency, GISELL, HSCT, immune dysregulation, liver, mTORC1, sirolimus

## Abstract

**Introduction:**

Biallelic loss-of-function mutations in the GTPase of immunity-associated protein 5 (*GIMAP5*) cause a severe syndrome characterized by altered immunity, lymphoproliferation, and progressive hepatopathy. This study aims to delineate the immunophenotypic signatures and clinical management of this deficiency by reporting the first Italian pediatric case alongside his mono-allelic carrier twin, integrated with a review of twenty previously published patients.

**Methods:**

We utilized trio-based clinical exome sequencing, flow cytometric immunophenotyping, protein expression profiling, and curated database analysis to evaluate the clinical trajectories.

**Results:**

The 11-year-old proband presented with autoimmune cytopenias, severe viral infections, and early biochemical liver anomalies. Genetic analysis identified compound heterozygous variants (p.Leu204Pro and p.Arg214Ter) in *GIMAP5* gene resulting in absent protein expression, alongside an expanded atypical memory B-cell population and T-cell exhaustion. Notably, his dizygotic twin harbored the heterozygous p.Leu204Pro variant and exhibited decreased protein expression coupled with a localized fibro-adipose vascular anomaly, but lacked immune defects.

**Discussion:**

This observation suggests complex genotype-phenotype interactions, raising the question of whether partial GIMAP5 deficiency might subtly predispose to localized vascular anomalies under specific environmental or epigenetic conditions, likely requiring multi-hit mechanisms. Therapeutically, the proband achieved sustained clinical remission of cytopenias through immunomodulation with the mTOR inhibitor sirolimus. These findings expand the phenotypic spectrum of this disorder, underscoring a dual role in immune and endothelial homeostasis. Furthermore, the successful application of sirolimus highlights the efficacy of precision medical management as a viable strategy to stabilize patients, allowing time to carefully weigh the risks and benefits of definitive interventions, such as hematopoietic stem cell transplantation.

## 1 Introduction

GTPase of immunity-associated protein 5 (GIMAP5) acts as a critical molecular checkpoint at the intersection of immune tolerance and vascular homeostasis. Predominantly expressed in T lymphocytes, Natural Killer (NK) cells, and endothelial cells, this evolutionarily conserved GTPase is indispensable for maintaining cellular quiescence and survival (1, 2). In preclinical models, GIMAP5 loss precipitates a catastrophic breakdown of homeostasis: it unleashes pathogenic effector programs and allergic inflammation via defective glycogen synthase kinase 3 beta (GSK3β) inactivation (1, 3) and accelerates cellular senescence through a dysregulated casein kinase 2 (CK2)–ceramide axis (4, 5).

Clinically, biallelic GIMAP5 deficiency manifests as a severe, pleiotropic syndrome recently framed as “GISELL disease” (GIMAP5 deficiency with Immune dysregulation, Splenomegaly, Enlarged Lymph nodes, Liver disease, and Lymphopenia) (4)and originally named “Noncirrhotic portal hypertension 2” (MIM#619463) in OMIM. Across the 20 cases described as of the end of 2025, the phenotype ranges from recurrent infections and autoimmunity to lymphoproliferation (6). However, hepatic involvement has emerged as a defining and often fatal feature. Unlike typical immune-mediated hepatopathies, GIMAP5 deficiency drives a distinct vascular remodeling characterized by nodular regenerative hyperplasia (NRH) and porto-sinusoidal vascular disorder (PSVD), frequently progressing to non-cirrhotic portal hypertension (2, 7, 8). Mechanistic evidence suggests this liver pathology is driven by the intrinsic “capillarization” of liver sinusoidal endothelial cells, challenging the assumption that the disease is solely hematopoietic in origin (2).

This complex pathophysiology creates a profound therapeutic dilemma with no established consensus. The current clinical decision-making process stands at a precarious crossroads between conservative precision medicine and definitive intervention, such as hematopoietic stem cell transplantation (HSCT) and/or liver transplant. Targeted therapies, particularly mTORC1 inhibitors like sirolimus, offer a compelling strategy to reverse the metabolic and senescent defects observed in patient lymphocytes (6). Conversely, HSCT promises immune reconstitution but poses significant mortality risks in patients with pre-existing vascular fragility and organ damage. Crucially, if the hepatic vascular defect is endothelial-intrinsic, HSCT may fail to halt the progression of portal hypertension, rendering the risks difficult to justify (2).

In this study, we report the first identified Italian case of GIMAP5 deficiency, presenting with early immune dysregulation and biochemical liver involvement. We describe the clinical trajectory of the pediatric proband alongside his dizygotic mono-allelic carrier twin, who is clinically and immunologically asymptomatic aside from an extensive vascular malformation of the lower limb, offering a unique comparative perspective on genotype-phenotype penetrance. By integrating this case with a comprehensive review of 20 previously published patients across eleven Eurasian kindreds, we aim to delineate distinct immunophenotypic signatures—specifically regarding the mTOR/pS6 pathway and other possible biomarker such as follicular helper T (Tfh) cells—and provide a critical analysis of the risk-benefit profile of targeted therapy versus HSCT in the management of this complex disorder.

## 2 Methods

### 2.1 Ethics and informed consent

All procedures performed in the study were in accordance with the ethical standards of the institutional research committee and with the 1964 Declaration of Helsinki and its later amendments. Informed consent, following standard ethical procedures, was obtained from the case-index patient and her parents. This study was approved by the Institutional Ethical Committee of Bambino Gesù Children Hospital. All subjects gave their informed consent to perform analysis.

### 2.2 Peripheral blood immunophenotyping

Flow cytometric analyses were performed on peripheral blood collected in EDTA tubes within 24 h from venipuncture. After red blood cells lysis with ammonium chloride, lymphocytes were washed, resuspended in PBS (Phosphate-buffered Saline, Sigma-Aldrich), and stained to identify T and B cell subsets with the following mouse anti-human antibodies: CD45RA (clone T6D11), CD3 (clone BW264/56), CD4 (clone REA263), TCR gamma-delta (11F2), CD8 (cloneREA734/HIT8a), CD19 (clone REA675/SJ25-C1) from Miltenyi Biotec, Bergisch Gladbach, Germany), CCR7 (clone 3D12; Thermofisher Scientific, Inc., MA,USA), CD27 (clone M-T271), TCR α-beta (clone T10B9), CD16 (clone 3G8), CD56 (clone MY31), CD21 (clone B-ly4), IgD (clone IA6-2), CD24 (clone ML5), CD38 (clone HIT2) from BD Biosciences, San Jose, CA, USA, and Goat F(ab)2 anti-Human IgM(μ) (Jackson ImmunoResearch Cambridge House, UK). Cells were incubated with the specific antibody cocktail for 30 min at room temperature, washed, and resuspended in PBS for acquisition. At least 50,000 events were acquired within the lymphocyte gate. Data were acquired on a FACSCanto II (BD Biosciences, San Jose, CA, USA) and analyzed with FlowJo software (Tree Star Inc., version 9.3.2, Ashland, OR, USA).

### 2.3 Peripheral regulatory T cells and circulating T follicular helper immunophenotyping

Isolated Peripheral Blood Mononuclear Cells (PBMC) were pre-treated with Fc-blocking reagent (Thermofisher Scientific Inc., MA, USA), then surface stained with specific antibodies for Treg identification and incubated for 30 min at room temperature. The Abs used were: CD4 (clone REA263; Miltenyi Biotec, Bergisch Gladbach, Germany), CD25(clone M-A251, BD Biosciences, San Jose, CA, USA), CD127 (ebioRDR5, Thermofisher Scientific, Inc., MA,USA), CD45RA (clone T6D11; Miltenyi Biotec, Bergisch Gladbach, Germany.) for the membrane staining and FoxP3 (clone PHC101, Thermo Fisher Scientific Inc., MA, USA) and Helios (clone 22F6, BioLegend San Diego, California, USA) for the nuclear transcription factors. Nuclear staining was performed with Foxp3 Transcription factor staining buffer set (Thermo Fisher Scientific Inc., MA, USA) according to manufacturer’s instructions. At least 200,000 CD4+ events within lymphocyte gate were acquired for each sample.

T helper follicular cells and their subsets were identified from fresh whole blood with the following monoclonal antibodies: CD4 (clone REA263), CXCR5(clone REA102/RF882), PD-1(clone REA1165), CXCR3(REA232/GO25H7 CD45RA (clone T6D11) from Miltenyi Biotec, Bergisch Gladbach, Germany and CCR6 (cloneG034E3) from BioLegend San Diego, California, USA),Erythrocytes were lysed with BD FACS Lysing Solution and leukocytes were washed with PBS. All samples were acquired with FACSCANTO II (BD Biosciences, San Jose, CA, USA) and analyzed with FlowJo software (Tree Star Inc, version 9.3.2).

### 2.4 NK cell degranulation assay

NK cell degranulation capacity was evaluated by measuring the surface expression of lysosomal-associated membrane protein-1 (LAMP-1/CD107a). PBMCs were cultured overnight in the presence of low-dose rhIL-15 (0.5 ng/mL) and then co-cultured with K562 target cells at a 1:1 effector-to-target ratio for 3 h at 37 °C and 5% CO_2_. The co-culture was performed in the presence of PE-conjugated anti-CD107a antibody. After incubation, cells were washed, stained with anti-CD3 and anti-CD56 antibodies, and analyzed by flow cytometry using a BD FACSVerse cytometer. NK cells were identified as CD3^−^CD56^+^ cells, and degranulation was quantified as the percentage of CD107a-positive cells within the NK cell population.

### 2.5 Ribosomal protein S6 phosphorylation flow cytometry assay

*Ex vivo* assessment of S6 Ser235/236 phosphorylation was performed on total blood using an antibody against phosphorylated S6 Ser235/236 (clone D57.2.2E, catalog 8520S, Cell Signaling Technology) and the PerFix EXPOSE kit, according to the manufacturer’s recommendations (Beckman Coulter). Briefly, 100 μl of freshly drawn blood was incubated in the presence of surface antibody for 10 minutes at 37 °C in a water bath. B cell and T cell surface markers were:CD19 (clone HIB19, catalog 2111030, Sony), CD21 (clone B-ly4, catalog 561374, BD), CD24 (clone ML5, catalog 555428, BD), CD57 (clone NK-1, catalog 555619, BD), CD3 (clone UCHT1, catalog 25-0038-42, BD), CD4 (clone VIT4, catalog 130-092- 373, Miltenyi Biotec), CD8 (clone BW135/80, catalog 130-096-902, Miltenyi Biotec). The reaction was stopped using buffer 1 and red blood cells were lysed with buffer 2 for 5 minutes at 37 °C. The samples were centrifuged, and the pellets underwent intracellular staining with buffer 3 for 1 hour. The cells were then washed with the dedicated buffer, resuspended, and analyzed with a FacsAriaII flow cytometer (Becton-Dickinson).

### 2.6 Trio-based CES analysis

NGS sequencing was performed on patient’s, parents’ and dizygotic twin’s genomic DNA on a NovaSeq6000 platform (Illumina). The reads were aligned to human genome build GRCh37/UCSC hg19. The Dragen pipeline and the Geneyx software LifeMap Sciences were respectively used for the variant calling and variant annotation. Global minor allele frequency (MAF) for analyzed variants was calculated according to Genome Aggregation Database (gnomAD). The variants were evaluated by VarSome (9) and categorized in accordance with the American College of Medical Genetics and Genomics (ACMG) recommendations (10).

### 2.7 Generation of T blasts

PBMC were isolated from patients and healthy controls (HDs) by density-gradient centrifugation with Ficoll-Paque PLUS (GE Healthcare) then. PBMC were cultured in complete medium (RPMI-1640, 10% FBS, glutamine, penicillin and streptomycin). To generate T blast, cells were plated in RPMI 10% FBS and activated with PHA (1 μg/ml, SIGMA) and IL2 (100 IU/ml, SIGMA).

### 2.8 Western blot

T-blast lines from patient, his brothers, his mother and HDs were incubating with complete JS 1× lysis buffer (50 mM Tris/HCl ph 8, 150 mM NaCl, 1.5 mM MgCl2, 5 mM EGTA, 1% Triton-X, 10% glycerol, 1 mM PMSF, aprotinin 1 mg/ml, leupeptin 1 mg/ml, pepstatin 1 mg/ml, 1 mM DTT) for 20 min/ice, clarified by centrifugation at 1600 rpm for 10 min at 4 °C to obtain total protein lysates. Lysates were size-fractionated by SDS-PAGE gel (12%) and then transferred to nitrocellulose membrane (Protran by Schleicher & Schuell-Bioscience, Dassel, Germany). The membrane was blocked in 5% milk at room temperature and then incubated GIMAP5 (#14108, Cell Signaling) 1:500 overnight. The day after we washed twice and incubated with goat anti-rabbit IgG (1 h, 1:5000, Cell Signaling) at 4 °C. The β-actin (1 h 1:5000, T9424, Sigma-Aldrich, Saint Louis, MO, USA) was used as housekeeping. The secondary goat anti-mouse antibody (Cell Signaling) was incubated 1:5000 1h at room temperature to detect the β-actin. Protein expression levels were analyzed with LiCor and calculated using standard densitometric analysis via Image studio Digits version 4.0, with the raw density of the GIMAP5 bands normalized against the β-actin housekeeping to determine relative fold-change.

### 2.9 Literature review and data collection

To comprehensively analyze the clinical and immunological spectrum of GIMAP5 deficiency, data from 20 previously published patients across 11 Eurasian kindreds were reviewed and integrated with our index case. All reported details of these patients were systematically documented and curated within GenIA (Genetic Immunology Advisor), a specialized database for inborn errors of immunity (IEIs). The curation process involved extracting and harmonizing diverse multidimensional data, which can be publicly accessed via the GenIA platform (https://geniadb.org/disease/info.php?id=6673).

Specific documentation and curation efforts focused on the following key parameters:

- Immunophenotypic and Functional Aberrations: Patient cohort data were curated to capture critical immunological signatures. This included documenting T-cell hyperactivation (such as increased CD25 and CD69 expression) and skewed cytokine production profiles, like elevated IFN-γ and IL-13. Additional extracted data encompassed the expansion of Th17 subsets, heightened markers of T-cell senescence (e.g., CD57 upregulation), increased baseline cell proliferation, and evidence of genomic stress, such as DNA damage markers.
- Therapeutic Interventions and Clinical Outcomes: Treatment modalities administered to the cohort were cataloged to evaluate patient responses. Clinical outcomes were meticulously categorized into qualitative tiers to enable a structured analysis. These tiers ranged from favorable outcomes (classified as “Excellent/Remission” or “Good”) to intermediate responses (“Mild”, “Moderate”, or “Unspecified”), down to unfavorable results (“Negative/Bad” or “Absent”).
- Data Visualization: The curated dataset facilitated the generation of detailed categorical overviews, heatmaps, and bar charts to visually summarize treatment efficacy and perform a critical risk-benefit analysis of targeted therapies versus HSCT.

## 3 Results

### 3.1 Case description and family history

Pt-2 is an 11-year-old male born from a dichorionic twin pregnancy at 39 weeks of gestation. He exhibited normal growth and development and had received routine immunizations. There was no reported parental consanguinity, and his dizygotic twin (Pt-3) and another sibling were clinically healthy. His past medical history was notable for a traumatic right occipital bone fracture at 6 years of age complicated by sigmoid sinus thrombosis, for which hematological investigations yielded normal results. He had no history of recurrent or severe infections prior to the index presentation.

The clinical onset occurred at 9 years of age, characterized by fever, pharyngitis, and laryngitis initially managed with outpatient amoxicillin-clavulanate. Pt-2 was subsequently hospitalized for pneumonia. Admission blood tests revealed leukopenia with profound neutropenia (nadir 130–240/mmc) and thrombocytopenia (27,000/mmc). Concurrently, he required metronidazole and ceftriaxone for periodontal abscess.

Microbiological investigation identified an acute Epstein-Barr Virus (EBV) and Parvovirus B19 coinfection. Bone marrow aspirate and biopsy demonstrated age-appropriate cellularity with dyshematopoietic features and reactive changes, supporting a peripheral origin for the cytopenias. High-dose intravenous immunoglobulin (IVIG) and corticosteroid pulses resulted in an initial recovery of platelet counts. During hospitalization, an episode of thrombophlebitis occurred at a previous venous access site and was treated with LMWH.

Right after disease onset, the patient had a period of persistent neutropenia and a single flare of thrombocytopenia (platelets 44,000/mmc) without symptoms and with spontaneous resolution. However, 7 months after the clinical onset, he was readmitted with severe hemorrhagic manifestations, including diffuse petechiae, gingival bleeding, epistaxis, and macroscopic hematuria. Laboratory findings revealed profound thrombocytopenia (platelets <2,000/mmc) and blood-loss anemia (hemoglobin nadir 7.5 g/dL). Autoimmunity work-up detected a direct antiglobulin test (DAT) positive for panagglutinating antibodies (anti-e specificity, titer 1:64) without biochemical hemolysis. The macroscopic hematuria was attributed to hemorrhagic cystitis; a bladder ultrasound revealed thickened, irregular, and lobulated walls. This was associated with JC polyomavirus infection, confirmed by high-level viruria rising to 6.8 × 10^6 copies/mL, although plasma PCR remained low or negative.

Systemic evaluation revealed hepatosplenomegaly with a coarse parenchymal echotexture on ultrasound and persistent hypertransaminasemia (AST 133 U/L, ALT 346 U/L).

Pt-3 (the dizygotic twin of Pt-2), presented at 10 years of age with a distinct, clinically silent phenotype unrelated to immune dysregulation. At 6 years of age, he had developed progressive gait difficulties, which led to further diagnostic evaluation. Imaging studies revealed a large pelvic mass measuring approximately 10.4 × 7.5 × 7.8 cm, compressing the rectum and bladder, displacing the femoral and iliac vessels, and extending through the obturator foramen into the inguinal region, reaching the anteromedial aspect of the proximal left thigh.

The lesion was incidentally characterized as a low-flow vascular malformation with lymphatic components. Both radiological findings and histopathological examination from an open biopsy were consistent with a diagnosis of fibro-adipose vascular anomaly (FAVA).

Due to the size and anatomical extension of the lesion, surgical and interventional management was required. Through follow-up, no laboratory abnormalities have been documented and no features suggestive of immune dysregulation have emerged.

Crucially, comprehensive clinical evaluation of the proband’s mother revealed a completely normal, asymptomatic phenotype. She exhibited no history of immune dysregulation or vascular malformations, and so did the proband’s father and his older brother (Pt-1).

### 3.2 Immunological findings

Peripheral blood immunophenotyping at age 10 revealed a quantitatively poor and functionally unbalanced lymphocyte compartment. Total CD3^+^ T cells were reduced (56.9%, 501/mmc) with marked CD4^+^ T lymphopenia (27.6%, 243/mmc) and skewing toward CD4+CD27+CD45RA- central (50.9%) and CD4+CD27-CD45RA- effector memory (30.7%) T cells, indicative of chronic immune activation/exhaustion. The frequencies of circulating CD4+CD45RO+CXCR5+ Tfh cells (cTfh) were slightly increased (10.2%).

CD19+ B-cell compartment was numerically expanded (40.3%, 355/mmc), with increased CD24++CD38++ transitional (21%) and mature naïve cells (51%). The frequency of CD27+IgD-IgM- memory B cells was relatively low at 10.7%, of which 80% were IgM and 20% were switched. Of particular relevance, approximately 15% of circulating B lymphocytes exhibited an “atypical memory” phenotype (CD19^+^CD38^−^CD24^−^CD21^−^CD27dim/neg), that is associated with persistent antigen exposure, immune exhaustion, or autoantibody-driven pathology.

CD16+CD56+ Natural killer cells were markedly reduced (1.8%, 16/mmc) as well as their degranulation and cytotoxicity capacity, consistent with impaired antiviral surveillance and potentially contributing to inadequate clearance of latent infections, although the test results may have been influenced by concomitant steroid therapy and the recent completion of a rituximab cycle.

Conversely, Pt-3 immunological evaluation at age 11 remained unremarkable, showing normal lymphocyte subsets, preserved T-/B-cell proliferation and B-cell maturation with protective antibody titers following vaccinations, including to pneumococcal vaccine.

All immunological data are reported in **Tables 1**, **2**.

**Table 1:** Pt-2’s immunological values.

Variable	Value^	Value^^	Value^^^	Normal range
**Lymphocytes (/mmc)**	1,200	**880** ↓	2,850	1,040-6,480
% Lymphocytes subsets				
CD3+CD45+	52.2	56.9	78.2	52.0-90.0
**CD19+CD45+**	**43.1 ↑**	**40.3 ↑**	16.9	7.0–24.0
**NK (CD3-CD16+CD56+)**	**3.6** ↓	**1.8** ↓	4.4	4.0-51.0
% T lymphocytes subsets				
CD3+CD4+	26.2	27.6	23.2	20.0-65.0
**CD3+CD4+CD27+CD45RA+ Naïve**	NA	**17.0** ↓	NA	37-97
CD3+CD4+CD27+CD45RA- Central memory	NA	50.9	NA	13-76
**CD3+CD4+CD27-CD45RA- Effector memory**	NA	**30.7 ↑**	NA	0.48-25
**CD3+CD4+CD27-CD45RA+ EMRA**	NA	**1.3** ↓	NA	9-63
**CD3+CD4+CD31+CD45RA+ RTE**	NA	**19.2** ↓	NA	31-81
CD4+CD25hiCD127lowFoxp3+ Treg cells	NA	4.0	NA	3.7 – 9.0 (25° - 75° p.ile)
**CD4+CD45RO+CXCR5+**	NA	**10.2 ↑**	NA	8.3 ± 1.8% of CD4^+*^
CD3+CD8+	19.8	23.8	**45 ↑**	14.0-40.0
CD3+TCRαβ	87.2	86.3	NA	39.0-92.0
TCRγδ	7.1	11.0	NA	2.0-17.0
CD3+CD4-CD8-TCRαβ+	0.8	1.0	NA	0.54-7.0
% B lymphocytes subsets				
**CD27+ Memory**	NA	**10.7**↓	**1.7↓**	13.3–47.9
- CD27+IgD+IgM+ Unswitched memory	NA	80.0	49.0	–
- CD27+IgD-IgM- Switched memory	NA	20.0	51.0	–
**CD27-IgD+IgM+ Naïve**	NA	**51.0** ↓	**82.6 ↑**	51.3–82.5
**CD24high CD38high Transitional**	NA	**21.0 ↑**	10.0	1.4–13.0
CD38++IgM- PlasmaBlasts	NA	2.3	4.5	0.6–6.5
**CD19** ^+^ **CD38** ^−^ **CD24** ^−^ **CD21** ^−^ **CD27dim/neg**	NA	**15.0 ↑**	**4.5 ↑**	–
Serum immunoglobulin levels				
**Serum IgG mg/dl**	**2,146 ↑**	**3,945 ↑**	**1,355 ↑**	595-1,310
**Serum IgM mg/dl**	**193 ↑**	154	67	31-179
Serum IgA mg/dl	154	**203 ↑**	109	53-200
Microbiological investigation				
**EBV viremia**	**50,309 copies/mL**	**1,755 copies/mL**	**<100 copies/mL**	–
**Parvovirus B19 viremia**	**1,225,700 copies/mL**	**311 copies/mL**	Negative	–

^Analysis performed 17 days after the second high-dose IVIG cycle and 4 days upon the second methylprednisolone bolus.

^^Analysis performed 2 days after high-dose IVIG and 1 day after the first methylprednisolone bolus, prior to rituximab.

^^^Analysis performed 11 months after initiating sirolimus and while on IVIG replacement therapy.

NA, Not available. B cell subsets from Piatosa B. et al. Cytometry part B (Clinical Cytometry) 2010 and Duchamp M. et al. Immunity, Inflammation and Disease 2014. T cell subsets from Garcia-Prat M. et al. Cytometry B Clin Cytom. 2019; Schatorje´ E. J. H. Clinical Immunology 2012and *Morita R. et al. Immunity. 2011 Jan 28;34(1):108-21. Serum Immunoglobulin concentrations from internal laboratory values. Values under the the lower limit of normal and over the upper limit of normal are reported in bold.

**Table 2:** Pt-3’s immunological values.

Variable	Value	Normal range
**Lymphocytes (/mmc)**	2,190	1,040-6,480
% Lymphocytes subsets		
CD3+CD45+	57.5	52.0-90.0
CD19+CD45+	12.0	7.0–24.0
NK (CD3-CD16+CD56+)	29.3	4.0-51.0
% T lymphocytes subsets		
CD3+CD4+	28.9	20.0-65.0
CD3+CD4+CD27+CD45RA+ Naïve	56.0	37-97
CD3+CD4+CD27+CD45RA- Central memory	33.6	13-76
CD3+CD4+CD27-CD45RA- Effector memory	10.2	0.48-25
**CD3+CD4+CD27-CD45RA+ EMRA**	**0.1**↓	9-63
CD3+CD4+CD31+CD45RA+ RTE	49.7	31-81
CD3+CD8+	22.3	14.0-40.0
% B lymphocytes subsets		
CD27+ Memory	27.2	13.3–47.9
- CD27+IgD+IgM+ Unswitched memory	54	–
- CD27+IgD-IgM- Switched memory	46	–
CD27-IgD+IgM+ Naïve	62.6	51.3–82.5
CD24high CD38high Transitional	3.4	1.4–13.0
**CD38++IgM- PlasmaBlasts**	**6.8 ↑**	0.6–6.5
Serum immunoglobulin levels		
Serum IgG mg/dl	1,040	595-1,310
Serum IgM mg/dl	155	31-179
**Serum IgA mg/dl**	**206 ↑**	53-200

B cell subsets from Piatosa B. et al. Cytometry part B (Clinical Cytometry) 2010 and Duchamp M. et al. Immunity, Inflammation and Disease 2014. T cell subsets from Garcia-Prat M. et al. Cytometry B Clin Cytom. 2019; Schatorje´ E. J. H. Clinical Immunology 2012and *Morita R. et al. Immunity. 2011 Jan 28;34(1):108-21. Serum Immunoglobulin concentrations from internal laboratory value. Values under the the lower limit of normal and over the upper limit of normal are reported in bold.

### 3.3 Genetic investigation

**Figure 1:**
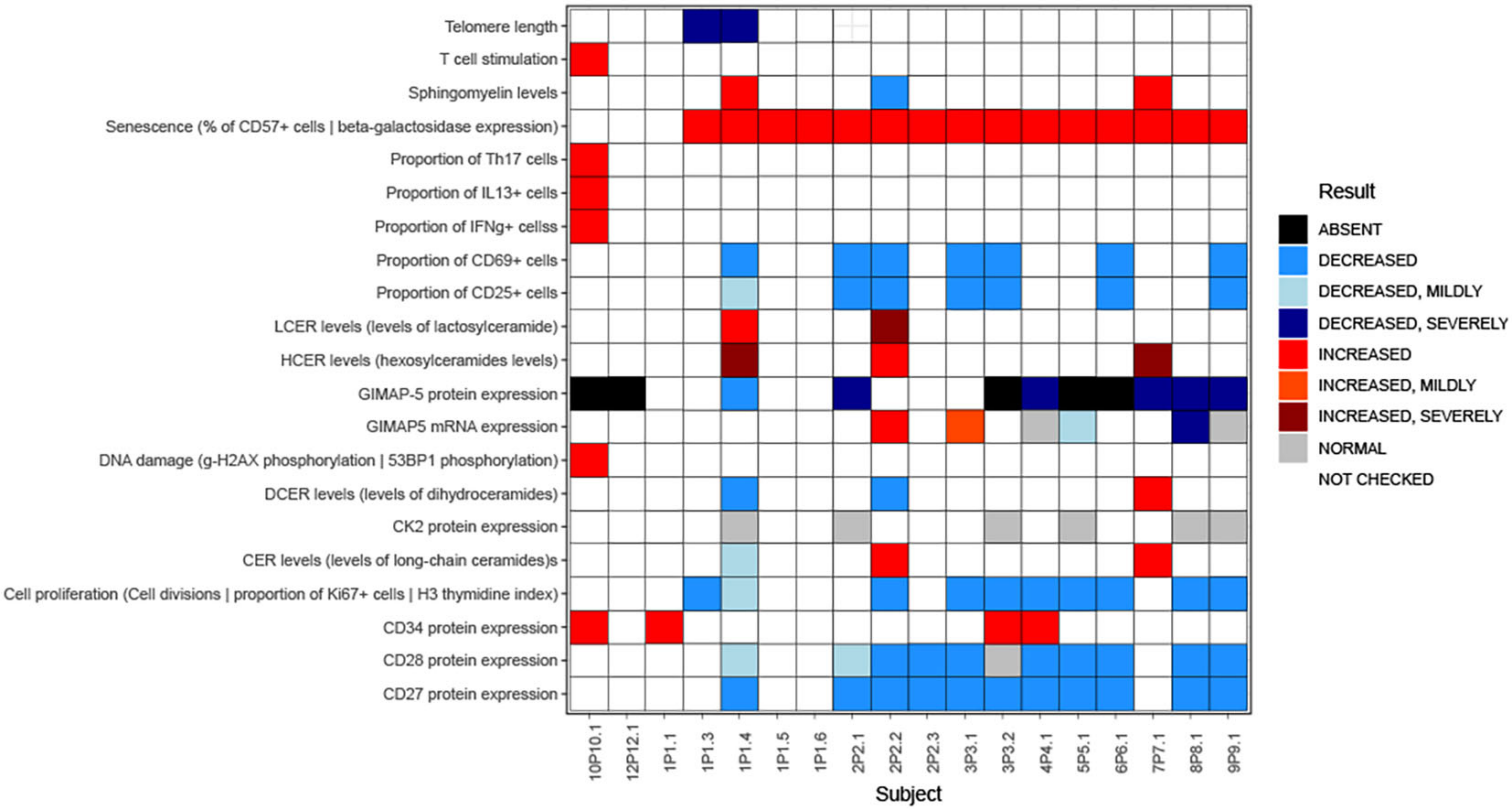
1A Family tree. 1B GIMAP5 protein expression by WB performed on T-blast derived from Pt-2, Pt-3, and HD showing complete absence and decreased expression in Pt-2 and Pt-3 respectively. 1C Densitometric analysis.

Trio-based CES sequencing analysis, performed by *in-silico* targeted gene panel relevant to the clinical indication, identified two rare compound heterozygous variants in Pt-2 in *GIMAP5* gene, c.611T>C and c.640C>T, resulting respectively in the missense substitution p.Leu204Pro (rs72650695) and the premature termination variant p.Arg214Ter (rs780355836). The missense variant c.611T>C (p.Leu204Pro) and the nonsense variant c.640C>T (p.Arg214Ter) were inherited maternally and paternally, respectively, and both have been previously described (1–4, 7). Subsequent genetic analysis by Sanger sequencing performed on Pt-3 showed that he only carried the c.611T>C (p.Leu204Pro) heterozygous maternal variant (**Figure 1A**), while his older brother (Pt-1) carried the c.640C>T (p.Arg214Ter) heterozygous paternal variant.

### 3.4 Laboratory findings

To evaluate the effect of the two variants on GIMAP5 protein expression, we performed western blot analysis on T-cell blasts derived from Pt-2, Pt-3 and an healthy donor (HD). The analysis showed a total loss of GIMAP5 protein expression in Pt-2 and a decreased expression (~2.5-fold) in Pt-3 compared to HD.

Mechanistically, the maternally inherited p.Leu204Pro substitution occurs within the highly conserved GTPase (G) domain of the protein. The introduction of a structurally rigid proline residue at this position is predicted to break helix α5 and disrupt its interaction with a loop containing W73. These events could cause major structural disruption to the critical alpha-helical packing of the G domain, resulting in severe thermal destabilization and protein misfolding. Consequently, this misfolding could expose hydrophobic surfaces that act as protein degradation signals, driving subsequent rapid proteasomal degradation (4, 6). To validate this, Western blot analysis on T-cell blasts from the proband, the twin, and both parents was performed. Densitometric quantification confirmed this destabilizing effect: both asymptomatic mother and father (heterozygous carriers of p.Leu204Pro and p.Arg214Ter, respectively) exhibited an intermediate reduction in GIMAP5 protein expression compared to the healthy donor (HD); the same goes for Pt-3. This moderate protein expression reduction affecting heterozygous carriers of p.Leu204Pro and p.Arg214Ter mirrored the findings by Park et al.

### 3.5 Therapeutic interventions and outcomes

Management of severe immune dysregulation required a multimodal approach for Pt-2. The acute hemorrhagic crisis due to thrombocytopenia relapse, occurring 7 months after the clinical onset (at 10.6 years of age), was treated with high-dose IVIG, 3 methylprednisolone pulses (30 mg/kg), and subsequent oral prednisone tapering. Due to refractory thrombocytopenia and suspected autoantibody production, B-cell depletion with rituximab (four doses at 375 mg/m2/weekly) was started concomitantly to the second methylprednisolone pulse.

Following the genetic diagnosis and recurrence of thrombocytopenia during steroid tapering, requiring additional high-dose IVIG alongside the thrombopoietin receptor agonist eltrombopag (max 50 mg/die), we initiated targeted immunomodulation with sirolimus (max 2.5 mg/die), a mTOR inhibitor, to control immune dysregulation. Although functional assays did not show constitutive mTORC1 hyperactivation, performed on steroid therapy (**Supplementary Figure 1**), sirolimus was selected based on the underlying pathophysiology of GIMAP5 deficiency (4, 6, 11). However, three months later, he experienced a Herpes Zoster (VZV) reactivation (viremia: 36,000 copies/mL) requiring hospitalization, temporary discontinuation of both sirolimus and steroids, and treatment with intravenous acyclovir and high-dose IVIG. This interruption of immunomodulation led to a transient relapse of severe thrombocytopenia (platelets 5,000/mmc) in the successfully treated with IVIG.

After the reintroduction of sirolimus and eltrombopag, combined with regular IVIG replacement (600 mg/kg/monthly), clinical stability was achieved. Platelet counts normalized in a month (range 311–477,000/mmc), and hemoglobin levels stabilized (>12 g/dL). Considering the stable and satisfactory platelet counts, a gradual tapering of eltrombopag was undertaken until its suspension.

JC virus monitoring showed clearance of viruria and resolution of viremia.

No brain MRI alterations were found. As of the last follow-up, Pt-2 maintains substantial clinical well-being with no further hemorrhagic or severe infectious episodes. Liver assessment demonstrates only mild persistent hypertransaminasemia with normal stiffness on elastography.

Pt-3, at age 11, is awaiting definitive surgical resection as the lesion’s size and vascularization made preoperative embolization unfeasible, according to interventional radiology and surgical consultants.

## 4 Discussion

Pt-2 is an 11-year-old boy who presented with severe immune dysregulation (autoimmune cytopenias, EBV viremia, and systemic inflammation) and biochemical signs of liver involvement. Genetic testing revealed compound heterozygous *GIMAP5* variants, a missense p.Leu204Pro and a nonsense p.Arg214Ter, already reported in prior GIMAP5 deficiency cases, but their combination in this patient expands the genotypic spectrum and marks the first Italian case of this disorder. Clinically, his phenotype aligns with the recently described GISELL syndrome (4). Interestingly, he experienced an atypical severe JC virus urinary infection with hemorrhagic cystitis, probably worsened by concomitant autoimmune thrombocytopenia previously triggered by viral infections. This severe viral manifestation broadens the clinical spectrum beyond what has been previously documented. In essence, this case underscores that biallelic *GIMAP5* mutations can manifest as a complex immuno-vascular syndrome early in childhood, adding new geographic and phenotypic insights to the spectrum of GIMAP5 deficiency.

Notably, the patient’s dizygotic twin (Pt-3) harbors the same maternal loss-of-function p.Leu204Pro variant without exhibiting immunological abnormalities. Interestingly, Pt-3’s sole clinical anomaly is an extensive vascular malformation of the lower limb. This finding challenges the established concept of “haplosufficiency” for this mutant allele (2, 4) and is rendered even more compelling by the decreased GIMAP5 protein expression (~2.5-fold) in Pt-3 compared to the HD (**Figures 1B, C**). Despite the compelling clinical and familial observations surrounding this genotype , the intrinsic pathogenicity of the maternally inherited GIMAP5 p.Leu204Pro (c.611T>C) variant warrants rigorous critical appraisal. While the substitution of a leucine with a conformationally rigid proline is hypothesized to disrupt the alpha-helical packing of the GTPase domain, population genetics present a highly nuanced paradigm. This missense alteration affects a residue lacking strict evolutionary conservation (PhyloP100: 3.20) and is present at a relatively high minor allele frequency of 0.00281 in the GnomAD database, which notably includes 11 seemingly healthy homozygotes. Consistent with a CADD score of 22, several in-silico algorithms predict a benign consequence, driving its conflicting pathogenicity classifications in ClinVar. Crucially, although previous functional studies and our own western blot data demonstrate diminished or absent GIMAP5 expression associated with this allele, this evidence must be interpreted cautiously. The single commercially available antibody for GIMAP5 targets a central epitope in close proximity to Leu204; thus, the apparent loss of protein could partially reflect abrogated antibody recognition of the mutated sequence rather than absolute proteasomal degradation. Clarifying whether this variant functions as a true null allele or simply an under-detected hypomorph remains essential for fully decoding the incomplete penetrance, multi-hit hypotheses, and variable immuno-vascular phenotypes observed in heterozygous carrier Pt-3.While decreased GIMAP5 protein expression (~2.5-fold) in Pt-3 could be coincidental, it raises the question of whether even partial GIMAP5 deficiency might subtly affect vascular development or endothelial function in some contexts (2). Indeed, it is possible that unfavorable modifier genes or epigenetic factors in the twin exacerbate the impact of haploinsufficiency, highlighting the complex genotype-phenotype interplay, as highlighted by Ciriaci et al, discussing the genetic predisposition to PSVD in the context of multi-hit hypotheses beyond GIMAP5 (8). While GIMAP5 deficiency unquestionably involves an intrinsic endothelial defect driving hepatic PSVD, the isolated thrombotic episodes observed in Pt-2 (such as the sigmoid sinus thrombosis following cranial trauma and the thrombophlebitis at a prior venous access site) were likely heavily influenced by environmental risk factors and direct mechanical trauma, rather than being solely attributable to his genetic status. Overall, the comparison between these brothers illustrates that a single functional *GIMAP5* allele is generally sufficient for immune homeostasis under normal conditions, but loss of both alleles (as in the proband) is required to produce the full GIMAP5 deficiency syndrome – emphasizing the role of environmental context and genetic modifiers in disease penetrance.

**Figure 2:**
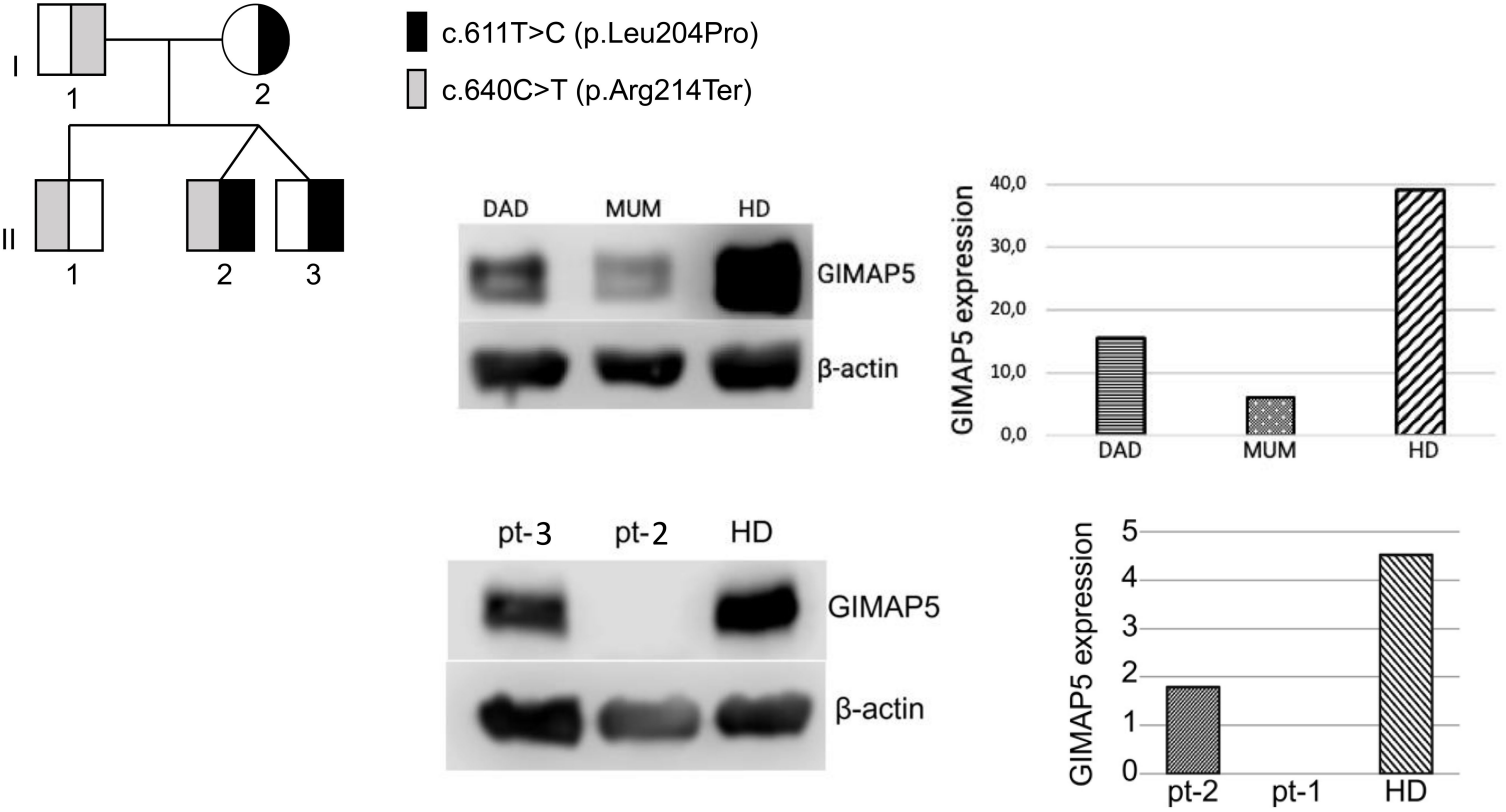
Immunophenotypic and functional aberrations in GIMAP5 deficiency. From patient cohort data. Key findings include T-cell hyperactivation (increased expression of activation markers like CD25 and CD69), skewed cytokine production profiles (e.g. elevated IFN-γ and IL-13 in patient T cells), an expanded Th17 subset, and heightened markers of T-cell senescence (such as CD57 upregulation). GIMAP5-deficient lymphocytes also show increased baseline proliferation and evidence of genomic stress (DNA damage markers). Our proband (Pt-2) is reported as 12P12.1 in the figure.

Including our patient, 21 individuals with GIMAP5 deficiency have been reported to date. All reported details of these patients can be found in GenIA (https://geniadb.org/disease/info.php?id=6673). Pt-2’s presentation is largely congruent with the core features observed in earlier cases (4). Almost all reported patients developed abnormal lymphoproliferation (20/21, 95%) and cytopenias, predominantly thrombocytopenia (16/21, 76%), often immune-mediated (11/21, 52%) or multilineage bone marrow failure (9/21, 43%), at a young age (1). Many also suffered from severe viral infections (6/21, 29%) – in particular, herpetic infections – reflecting the crucial role of GIMAP5 in T/NK-cell antiviral immunity (4). Pt-2’s high EBV load and inability to clear the virus is therefore a common theme. Likewise, B- and T-cell dysregulation is frequently seen in form of hypogammaglobulinemia (10/21, 48%), or paradoxical hyper-IgM (2/21, 9%), and abnormal T cell subset distribution (17/21, 81%), often in form of an enrichment of activated, effector memory T-cell subsets (**Figure 2**), as reported in GIMAP5 deficient mice (11, 12). In Pt-2, we documented a similar pattern – elevated CD4^+^ effector-memory T cells and circulating CD4^+^CXCR5^+^ Tfh cells (slightly above age norms), alongside an expanded “atypical” memory B-cell population (~15% of B cells), which may be associated with chronic antigen exposure or exhaustion — a feature that has not been explicitly highlighted in prior GIMAP5-deficient cohorts. Of note, this atypical memory B-cell subset demonstrated a decreasing trend, representing 4.5% of the total B-cell compartment at the last follow-up. This reduction occurred concurrently with the sustained clinical remission of thrombocytopenia, suggesting that the rapid decrease in this peculiar B-cell subset correlates directly with the pharmacological suppression of chronic inflammation via mTOR inhibition. While strictly anecdotal in this single subject, this observation mirrors the mTORC1-dependent expansion of atypical memory B cells seen in other systemic rheumatic conditions (13) and in settings of chronic antigen exposure, such as malaria (14) and HIV (15). Consequently, tracking this subset warrants longitudinal investigation in larger GIMAP5-deficient cohorts to determine its broader utility as a correlative parameter of treatment efficacy. Another hallmark of GIMAP5 deficiency is progressive liver involvement. The 86% (18/21) of published patients developed hepatic manifestations ranging from persistently elevated transaminases (9/21, 43%) to nodular regenerative hyperplasia (5/21, 24%), portal hypertension (13/21, 62%) and abnormality of the hepatic vasculature (16/20, 76%) (2, 4, 7). Pt-2’s liver disease is currently mild (transaminitis without fibrosis or portal hypertension), but his course likely represents an early stage of the distinctive vasculopathy described in GIMAP5 deficiency. Furthermore, the clinical evaluation of the proband’s mother—who harbors the identical heterozygous p.Leu204Pro variant—revealed a completely asymptomatic phenotype. This profound clinical discordance strongly implies that the twin’s peripheral vascular anomaly may be a sporadic, anecdotal occurrence, potentially driven by a localized somatic second hit, rather than a direct, highly penetrant consequence of partial GIMAP5 deficiency alone (2). In summary, our case both mirrors the shared features of previously reported cases – cytopenias, EBV susceptibility, lymphoproliferation, and B-cell/T-cell subset imbalances – and provides novel insights such as atypical memory B-cell expansion and a heterozygous phenotypic variant, thereby enriching the clinical and immunological portrait of this IEI.

Consistent with these trends, Pt-2’s T cells exhibited an activated, proliferation-prone phenotype; In murine models and some human patients, GIMAP5 loss leads to constitutive mTORC1 hyperactivation in T cells (reflected by high baseline phospho-S6 levels) (6). However, Pt-2’s T cells did not show elevated phospho-S6 during initial functional assays, likely because concurrent high-dose corticosteroids blunted the mTOR signaling axis at the time of testing. Nevertheless, given the known biology of GIMAP5, it is assumed that mTOR dysregulation was an underlying feature once steroids were tapered. Indeed, the therapeutic response to sirolimus (an mTORC1 inhibitor) in Pt-2 supports the idea that mTOR-driven immune activation was clinically relevant despite the normal pS6 lab result (6, 11). We also observed a slight expansion of circulating Tfh cells in the proband, which aligns with reports of aberrant Tfh: Treg ratios in GIMAP5 deficiency in mice (16). An imbalance favoring Tfh cells may contribute to B-cell dysregulation and autoantibody production seen in these patients. Overall, the immunophenotype of our patient, characterized by T-cell activation, Tfh skewing, NK cell dysfunction, and aberrant B-cell maturation, fits within the expanding spectrum of GIMAP5 deficiency, while also highlighting how treatment status can influence the detection of these abnormalities.

**Figure 3:**
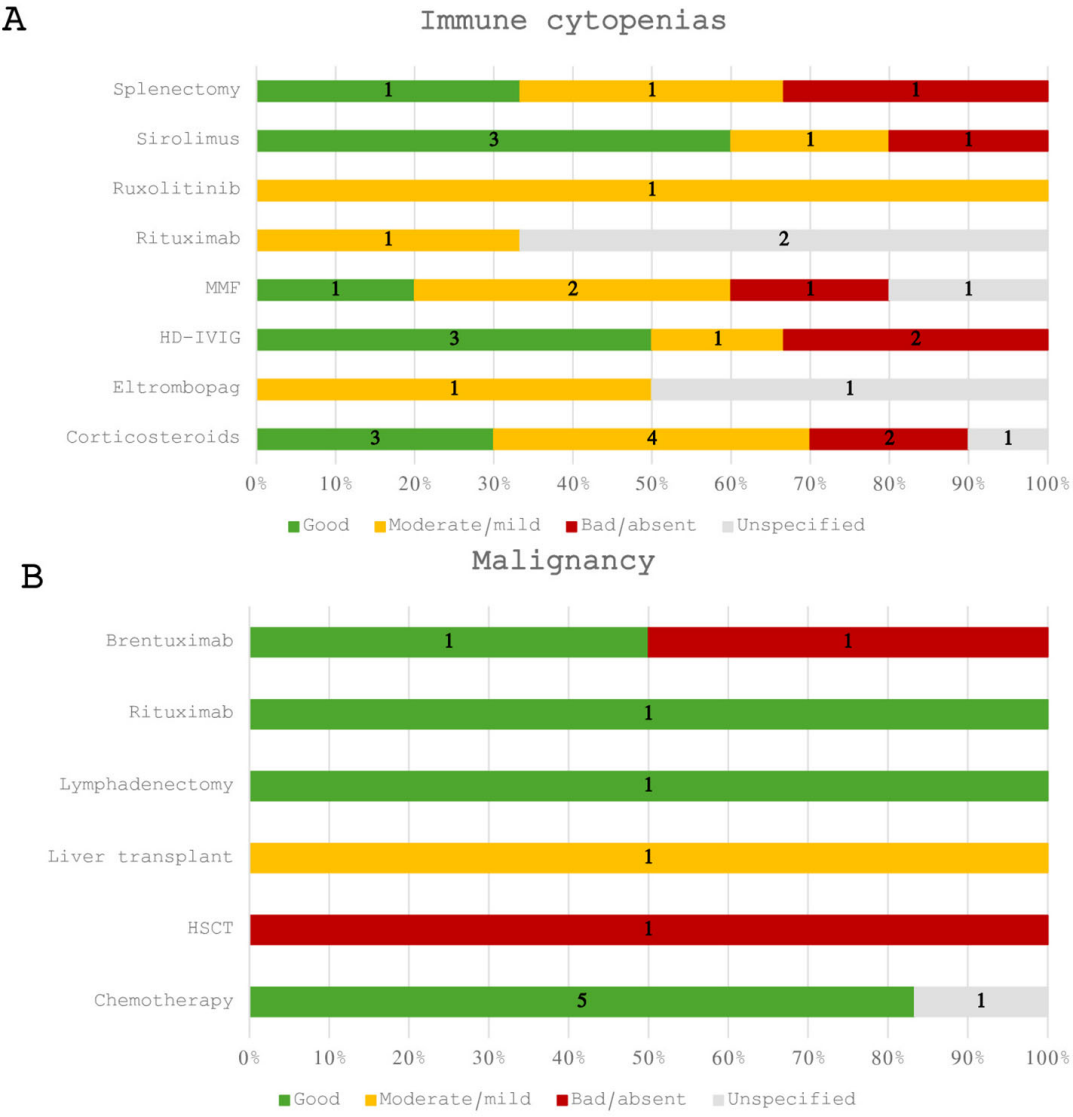
Treatment modalities and patient responses in reported GIMAP5 deficiency cases. Bars chart summarizes clinical responses to various therapies across published GIMAP5-deficient patients (including the present case, Pt-2). For each intervention, the colored segments indicate the number of patients achieving a good response (green), moderate/mild response (yellow), bad/absent response (red), gray or an unspecified response (grey). **(A)** represents treatment for immune cytopenias and **(B)** treatments for malignancy.

In the absence of established guidelines, management of GIMAP5 deficiency has been largely empirical, aiming to control autoimmunity and hyperinflammation while balancing infection risks. As illustrated in **Figures 3A, B**, and **Supplementary Figures 2**, **3**, corticosteroids and high-dose IVIG are the most frequently employed first-line therapies, yielding at least temporary improvement in cytopenias or inflammatory symptoms in the majority of patients (44). Pt-2’s initial treatment mirrored this approach: high-dose IVIG and methylprednisolone pulses produced a transient rise in platelets, but the effect waned, and disease activity rebounded during steroid tapering. Rituximab was added to target presumed autoantibody-mediated platelet destruction; this achieved a degree of B-cell depletion and was associated with some stabilization of platelet counts, but not a durable remission. He also received eltrombopag as an adjunct to support platelet production. Despite these measures, the proband had recurring severe thrombocytopenia, prompting a pivot in therapy. Following the genetic diagnosis of GIMAP5 deficiency, sirolimus (rapamycin) has been started as a targeted immunomodulatory therapy. Notably, sirolimus was chosen based on the mechanistic rationale from murine models and prior patient reports (4). Encouragingly, Pt-2’s cytopenias entered a sustained remission on sirolimus (with platelet counts normalizing and anemia resolving), and his splenomegaly and inflammatory markers improved, consistent with the “good” responses to mTOR inhibition documented in other cases (4), although prolonged immunosuppression is associated with infectious complications. Indeed, while on sirolimus therapy Pt-2 developed an episode of herpes zoster requiring hospitalization, antiviral therapy, and temporary suspension of immunosuppression. Zoster reactivation is a known risk in patients on immunosuppressants, including mTOR inhibitors (17), and its occurrence in our case underscores the need for evaluation of vigilant prophylaxis (e.g. VZV vaccination or acyclovir prophylaxis) when instituting targeted therapies. Once the acute infection resolved, sirolimus was successfully reintroduced (along with monthly IVIG for passive immunity), and the patient has maintained clinical stability over the subsequent 12 months. This outcome highlights a central therapeutic dilemma: targeted medical therapy can effectively control the immune dysregulation in GIMAP5 deficiency, but it requires long-term immunosuppression with attendant risks, whereas HSCT could theoretically restore the hematopoietic compartment but carries its own risks and its efficacy is uncertain on all manifestations.

In our literature review, only a few GIMAP5-deficient patients have undergone HSCT, reflecting hesitancy due to the high-risk profile (2). Among the 20 published cases, outcomes of HSCT have been mixed – there is at least one report of transplant-related mortality (4), and other cases where HSCT failed to reverse progressive liver disease (2). GIMAP5 deficiency often involves a vicious cycle of immune-mediated damage and intrinsic endothelial pathology, and HSCT addresses only the hematopoietic component. If the liver’s sinusoidal endothelium lacks GIMAP5 (a defect not corrected by bone marrow transplantation), portal hypertension may continue to worsen even after a technically “successful” HSCT (2). This concern has been raised by Drzewiecki et al., who argue that transplant in GIMAP5 deficiency might not halt – and could even exacerbate – the unique hepatic vasculopathy, especially in patients who already have significant portal hypertension and fragile vasculature (2). On the other hand, HSCT offers the only prospect of definitive cure of the immune dysfunction, and could be justified in a patient with life-threatening disease that is unresponsive to medical therapy. Pt-2’s clinical course exemplifies this careful risk-benefit analysis. At presentation, he had serious but reversible complications (autoimmune cytopenias, infections) that responded to immunosuppressive therapy, and no irreversible organ damage. We therefore elected to pursue a conservative, targeted-treatment approach and defer HSCT. Over nearly 12 months of follow-up on sirolimus (with adjunct therapies), he has remained in remission from cytopenias, with improving immune parameters and only mild liver enzyme elevation. This favorable outcome suggests that, at least in some cases, long-term medical management can successfully mitigate disease manifestations. Nevertheless, close monitoring for any signs of liver disease progression or malignancy is strictly indicated, as such developments would prompt a rapid re-evaluation for HSCT. As more cases are reported, it will be important to identify prognostic markers – for instance, severity of early liver involvement or refractory cytopenias – that could guide clinicians on when to escalate to HSCT versus continue with medical therapy (4). For now, our experience supports an initial attempt of mTOR inhibition in GIMAP5 deficiency, especially for patients without advanced liver fibrosis, with HSCT reserved as a rescue for those who fail to stabilize on conservative measures.

When HSCT is pursued in this specific IEI, identifying the optimal timing for the procedure is critical; ideally, complete control of the underlying immune dysregulation should be achieved prior to transplantation to maximize the chances of a successful outcome. Furthermore, the selection of the pre-conditioning regimen is of paramount importance (18). Because GIMAP5 deficiency intrinsically drives the capillarization of liver sinusoidal endothelial cells and subsequent PSVD, these patients possess extreme baseline endothelial fragility. The application of standard myeloablative conditioning (MAC) regimens utilizing heavy alkylating agents (e.g., high-dose busulfan) carries an unacceptably high risk of triggering fatal Sinusoidal Obstruction Syndrome in the liver. Consequently, recent literature strongly advocates for the use of Reduced Toxicity Conditioning regimens for patients with pre-existing PSVD. A backbone consisting of treosulfan and fludarabine is highly preferable in this context. Treosulfan, while highly myeloablative and capable of securing robust donor chimerism, demonstrates a vastly superior hepatic safety profile compared to busulfan, minimizing further toxic insult to the compromised sinusoidal endothelium. Ultimately, however, every final therapeutic strategy must be highly individualized, remaining heavily dependent on the availability of an optimal donor (such as a matched sibling or matched unrelated donor, the chosen stem cell source, and the patient’s precise clinical condition at the time of the transplant (19, 20).

This case report has several limitations that warrant acknowledgement. First, as a single-patient observation, any therapeutic or mechanistic insights must be interpreted with caution. GIMAP5 deficiency is a heterogeneous disease, and the proband’s particular genotype and environmental exposures (e.g. EBV, JC virus) likely influenced his phenotype in ways that may not generalize to all patients. Second, some immunological analyses were performed during ongoing treatments (IVIG, corticosteroids, rituximab), which undoubtedly confounded some results. For example, B-cell depletion therapy and steroid use can mask mTOR and pS6 hyperactivity – thus, some immune abnormalities might have been underappreciated in our tests. Third, there may be ethnic and genetic background factors affecting disease expression. The majority of published GIMAP5-deficient kindreds, including ours, are of Eurasian origin (4, 7); subtle differences in genetic modifiers or access to care could influence outcomes, meaning the efficacy of sirolimus may not be universally replicable. Nevertheless, the clinical response observed in this case, together with the demonstrated efficacy of sirolimus in experimental animal models, strongly supports its therapeutic value. Furthermore, considering that mTOR pathway dysregulation is a primary pathomechanism in GIMAP5 deficiency, this paves the way for exploring additional targeted therapies that modulate this axis, such as leniolisib, which proved beneficial in patients with Activated Phosphoinositide 3-Kinase Delta Syndrome (21) and in murine autoimmune lymphoproliferative syndrome (22).

## 5 Conclusion

Despite its limitations, this report expands the clinical spectrum of GIMAP5 deficiency (GISELL syndrome), highlighting novel features such as atypical viral susceptibility (e.g., JC virus hemorrhagic cystitis). Early genetic diagnosis proved pivotal, enabling the anticipation of vascular complications like portal hypertension, the screening of relatives, and the successful implementation of mechanism-based mTOR inhibition. Consequently, we strongly advocate for the inclusion of *GIMAP5* in diagnostic gene panels for pediatric immune dysregulation, particularly when accompanied by hepatic anomalies. Moving forward, international registries will be essential to systematically compare the long-term safety and efficacy of targeted pharmacotherapies (e.g., sirolimus or JAK inhibitors) versus HSCT. Ultimately, this case illustrates that tailored medical management can achieve meaningful clinical stability, effectively buying critical time to carefully weigh the risks and benefits of definitive interventions—such as HSCT or emerging gene therapies—in this complex immuno-metabolic disorder (7).

## Data Availability

The original contributions presented in the study are included in the article/**Supplementary Material**. Further inquiries can be directed to the corresponding author/s.

## Supplementary material

Supplementary Figure 1 Ribosomal protein S6 phosphorylation flow cytometry assay.

Supplementary Figure 2 Treatment modalities and patient responses in reported GIMAP5 deficiency cases. Bars chart summarizes clinical responses to various therapies across published GIMAP5-deficient patients (including the present case, Pt-21). For each intervention, the colored segments indicate the number of patients achieving an excellent/remission-level response (dark purple), good partial response (dark blue), mild or transient improvement (light blue), no significant effect (gray), or a negative/adverse outcome (red/pink). Interventions are ranked roughly by usage frequency.

Supplementary Figure 3 Heatmap summary of therapeutic responses in GIMAP5-deficient patients. The figure displays a categorical overview of clinical outcomes (Response) associated with various treatment modalities (y-axis) administered to individual subjects within the cohort (x-axis). Response quality is color-coded: dark blue shades represent favorable outcomes (Excellent/Remission, Good); light blue shades denote intermediate or partial responses (Mild, Moderate, Unspecified); and red/pink tones indicate unfavorable outcomes (Negative/Bad, Absent). White cells indicate unreported data or interventions not applied.
