# Supplementary figures and images for: Unexpected genetically determined immune dysregulation with liver involvement: GIMAP5 therapeutic dilemmas between targeted therapy and HSCT
Source: Front Immunol. 2026 Jun 10;17:1820281. doi: 10.3389/fimmu.2026.1820281 (PMC13290706; doi:10.3389/fimmu.2026.1820281)

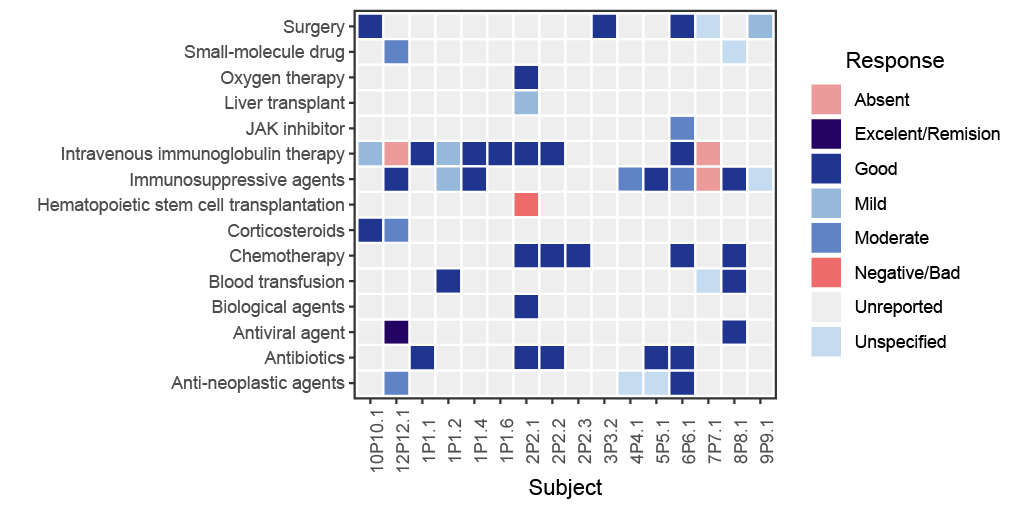

Supplement: Supplementary Figure 1 — Ribosomal protein S6 phosphorylation flow cytometry assay. [file Image1.tiff]

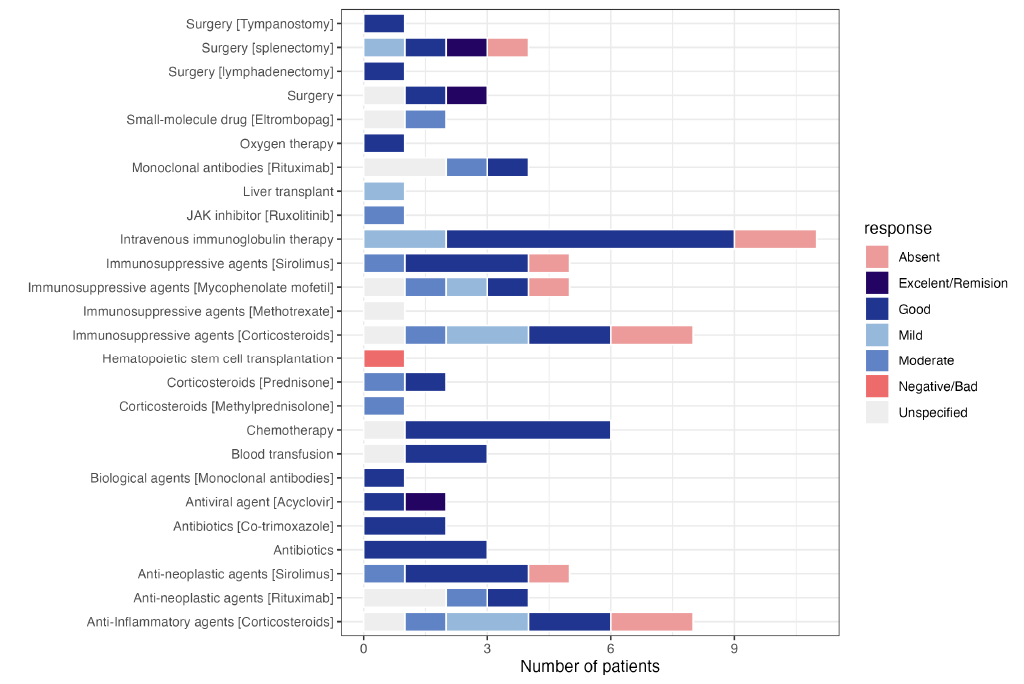

Supplement: Supplementary Figure 2 — Treatment modalities and patient responses in reported GIMAP5 deficiency cases. Bars chart summarizes clinical responses to various therapies across published GIMAP5-deficient patients (including the present case, Pt-21). For each intervention, the colored segments indicate the number of patients achieving an excellent/remission-level response (dark purple), good partial response (dark blue), mild or transient improvement (light blue), no significant effect (gray), or a negative/adverse outcome (red/pink). Interventions are ranked roughly by usage frequency. [file Image2.tiff]

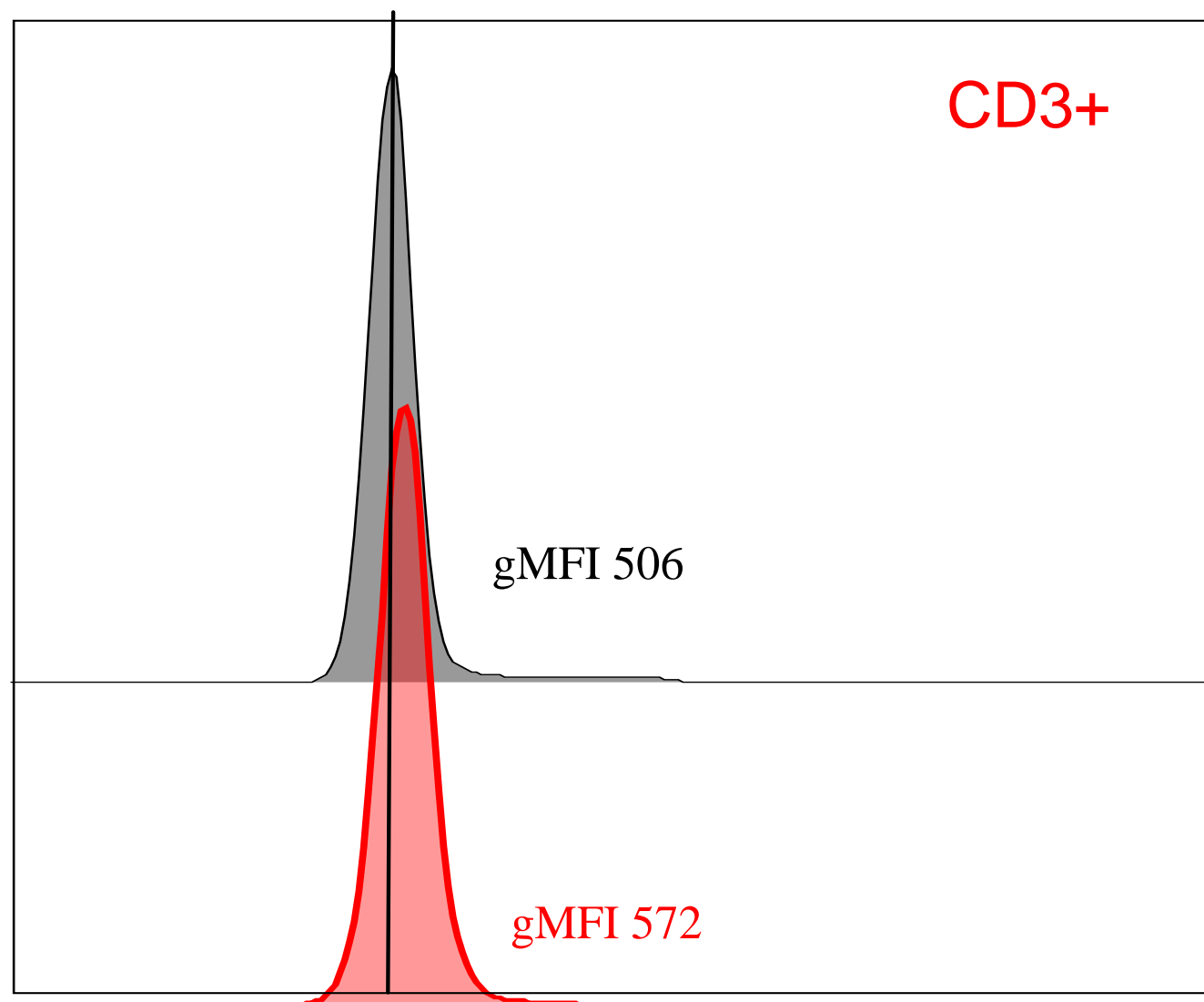

Supplement: Supplementary Figure 3 — Heatmap summary of therapeutic responses in GIMAP5-deficient patients. The figure displays a categorical overview of clinical outcomes (Response) associated with various treatment modalities (y-axis) administered to individual subjects within the cohort (x-axis). Response quality is color-coded: dark blue shades represent favorable outcomes (Excellent/Remission, Good); light blue shades denote intermediate or partial responses (Mild, Moderate, Unspecified); and red/pink tones indicate unfavorable outcomes (Negative/Bad, Absent). White cells indicate unreported data or interventions not applied. [file DataSheet1.pdf]
